# ASICs Do Not Play a Role in Maintaining Hyperalgesia Induced by Repeated Intramuscular Acid Injections

**DOI:** 10.1155/2012/817347

**Published:** 2011-12-08

**Authors:** Mamta Gautam, Christopher J. Benson, Jon D. Ranier, Alan R. Light, Kathleen A. Sluka

**Affiliations:** ^1^Graduate Program in Physical and Rehabilitation Science, University of Iowa, 1-252 MEB, Iowa City, IA 52242, USA; ^2^Department of Internal Medicine, University of Iowa, E315 GH, Iowa City, IA 52242, USA; ^3^Department of Chemistry, University of Utah, 315 S 1400 E RM 2020, Salt Lake City, UT 84112, USA; ^4^Department of Anesthesia, University of Utah, 30 N 1900 E RM 3C444, Salt Lake City, UT 84132, USA

## Abstract

Repeated intramuscular acid injections produce long-lasting mechanical hyperalgesia that depends on activation of ASICs. The present study investigated if pH-activated currents in sensory neurons innervating muscle were altered in response to repeated acid injections, and if blockade of ASICs reverses existing hyperalgesia. In muscle sensory neurons, the mean acid-evoked current amplitudes and the biophysical properties of the ASIC-like currents were unchanged following acidic saline injections when compared to neutral pH saline injections or uninjected controls. Moreover, increased mechanical sensitivity of the muscle and paw after the second acid injection was unaffected by local blockade of ASICs (A-317567) in the muscle. As a control, electron microscopic analysis showed that the tibial nerve was undamaged after acid injections. Our previous studies demonstrated that ASICs are important in the development of hyperalgesia to repeated acid injections. However, the current data suggest that ASICs are not involved in maintaining hyperalgesia to repeated intramuscular acid injections.

## 1. Introduction

Acid Sensing Ion Channels (ASICs) are found in peripheral neurons and play a significant role in modulation of nociceptive behavior following insult to muscle or joint. Decreases in pH activate ASICs, directly activate nociceptors, and produce pain in humans [1–4]. Four genes encoding six ASIC subunits (ASIC1a, ASIC1b, ASIC2a, ASIC2b, ASIC3, ASIC4) have been identified. Functional ASICs form as homotrimers or heterotrimers of three ASIC subunits, and in mouse DRG neurons the channels primarily exist as heteromers [5, 6]. Of the different isoforms, ASIC3 is found predominately in peripheral sensory neurons and has been shown to play a significant role in nociception. 

After inflammation of the muscle or joint, there is a reduction in cutaneous secondary hyperalgesia of the paw in ASIC3−/− mice and a reduction in primary muscle hyperalgesia in ASIC1−/− mice [7–10]. Restoration of ASIC3 expression in primary afferent fibers innervating muscle of ASIC3−/− mice rescues mechanical hyperalgesia after muscle inflammation [7], suggesting a significant role for peripheral ASIC3 in inflammatory hyperalgesia from muscle. In contrast, cutaneous inflammatory hyperalgesia is unchanged or even enhanced in ASIC3−/− mice [11–13]. However, peripheral blockade of ASIC3 at the time of injection or siRNA knockdown of ASIC3 in DRG prevents the development of cutaneous inflammatory hyperalgesia in rats [14]. Inflammation also induces an increased mRNA (ASIC1 and ASIC3) and protein expression (ASIC3) in DRG and increased protein expression in peripheral terminals of nociceptors [8–10, 15–18]. This enhanced expression in DRG is manifested as an increased responsiveness to acidic pH [19]. Once developed, inflammatory hyperalgesia is reversed by blockade of ASICs nonselectively, or by selective blockade of ASIC3 intrathecally [10, 20, 21]. Together these data suggest an important role of ASIC1 and ASIC3 in the development and the maintenance of inflammatory musculoskeletal pain. 

To model noninflammatory chronic muscle pain we developed a model induced by two injections of acidic saline, 5 days apart, into the gastrocnemius muscle [22]. Like wild-type mice, ASIC1−/− mice still develop secondary mechanical hyperalgesia of the paw; however, this response was completely abrogated in ASIC3−/− mice [23]. In addition, blockade of ASICs during the second acid injection with amiloride prevents the development of secondary hyperalgesia 24 hours later [23]. These data from our laboratory support that activation of ASICs, in particular ASIC3, is important for the development of secondary hyperalgesia in response to repeated acid injections. However, it is not known if there are changes in ASICs after the development of hyperalgesia in this model, or if ASICs in muscle afferents are important for maintaining the hyperalgesia. We therefore examined the properties of ASIC-like currents from retrogradely labeled muscle sensory neurons, and the effects of antagonism of ASICs on primary (muscle) and secondary (cutaneous) hyperalgesia 24 hours after the second injection of acidic saline. 

## 2. Materials and Methods

### 2.1. Animals

C57BL/6 male mice (age 2–4 months; n58) (Jackson Laboratories, Bar Harbour, Maine) were used in these studies. The Animal Care and Use Committee at the University of Iowa approved all experiments (ACURF#0908193).

### 2.2. Labeling of Muscle Sensory Neurons

Sensory neurons innervating muscle were fluorescently labeled using the retrograde tracer DiI (1,1-dioctadecyl-3,3,3,3 tetramethylindocarbocyanine perchlorate; 17 mg/mL dissolved in 20% v/v ethanol and suspended in 80% v/v sterile saline). Animals were anesthetized with 2–5% isoflurane, a small incision was made in skin over the left gastrocnemious muscle, and 10 *μ*L DiI was injected into the left gastrocnemius muscle as previously described (*n* = 30) [18]. After injection, saline-soaked sterile gauze was placed on the open incision for 10 minutes to prevent the dye from leaking to the overlying skin. The skin was then sutured closed and mice were allowed to recover for approximately 2 weeks. 

### 2.3. Intramuscular Acid Injections

For recording experiments, mice were injected 2 weeks after DiI injection into the same gastrocnemius muscle with 20 *μ*L of pH 4.0 (*n* = 10) or pH 7.2 (*n* = 11) saline while deeply anesthetized with isoflurane (5%). In approximately half the animals a second 20 *μ*L of injection of pH 4.0 (*n* = 6) or pH 7.2 (*n* = 6) saline was reinjected into the gastrocnemius muscle 5 days later. 24 hours after a single injection of saline, or 24 hours after a second injection of saline, mice were euthanized and the L4-L6 DRG neurons were isolated and cultured. For behavioral experiments, mice received an initial injection of 20 *μ*L of pH 4.0 (*n* = 24) while deeply anesthetized with isoflurane (5%), immediately after baseline behavioral testing. A second injection was repeated 5 days later in all mice. 

### 2.4. Isolation of DRG Neurons

The ipsilateral L4-L6 DRGs were collected and dissociated as previously described [6]. DRGs were treated with papain and collagenase/dispase and then gently triturated to isolate neurons. Neuron suspensions were then plated on 35 mm Petri dishes coated with poly L-lysine and laminin. Cells were cultured in F12 medium supplemented with 10% heat inactivated serum, penicillin-streptomycin, and 50 ng/mL NGF. 24 hours after plating we examined cells with whole-cell patch-clamp. 

### 2.5. Electrophysiology of Cultured DRG Neurons

Whole-cell patch-clamp recordings of DiI labeled DRG neurons were performed at room temperature at a holding potential of −70 mV. Currents were filtered at 1 kHz and sampled at 2 kHz using the Axopatch 200B amplifier, Digidata 1200, and Clampex 8.2 (Axon instruments, Union city, CA). Micropipettes (3–5 MΩ) were filled with internal solution (mM): 100 KCl, 10 EGTA, 40 Hepes, 5 MgCl_2_, pH 7.4 with KOH. External solutions contained (mM) 120 NaCl, 5 KCl, 1 MgCl_2_, 2 CaCl_2_, 10 HEPES, and 10 MES; pH was adjusted with tetramethylammonium hydroxide; osmolarity was adjusted with tetramethylammonium chloride. Extracellular pH solutions ((pH 7.4 (control)), 6.8, 6.5, 6.0, 5.0) were used to study ASIC currents. Whole cell capacitance was compensated and recorded. Solutions with different pH were applied directly to the cell by using a perfusion system BPS 8 (ALA scientific, Westbury NY), which was controlled by Digidata 1200 and Clampex 8 software (pClamp8). 

To measure pH dose responses, pH currents activated by pH 5, 6, 6.5, and 6.8 solutions were normalized to pH 5 induced currents. Time constants for desensitization were measured from single exponential fit to the falling phase of the current evoked by pH application. The time course of recovery from desensitization was measured by completely desensitizing the ASIC current at pH 6 by a long desensitizing pulse followed by bathing in pH 7.4 for a defined time followed by a second stimulation at pH 6. Recovery is percentage of recovery of current evoked by second pulse by first pulse. 

### 2.6. Behavioral Assessment

Mice were given one dose of 0.025 *μ*mol A-317567 (C-(6-[2-(1-isopropyl-2-methyl-1,2,3,4-tetrahydro-isoquinolin-7-yl)-cyclopropyl]-naphthalen-2-yl)-methanediamine) (10 *μ*L) injected into the left gastrocnemius muscle 24 hours after induction of hyperalgesia. This dose was based on our prior study which showed a reduction in pain-behaviors after muscle inflammation [10]. Separate mice were used to test behavioral sensitivity. Muscle sensitivity was tested as follows: before the first injection of the muscle, before the second injection of the muscle, 24 hours after the second injection, and 15 minutes after A-317567 injection. A-317567 was injected intramuscularly immediately after the 24 hours behavioral test. C57BL/6 mice were acclimated for 2 days before testing for muscle sensitivity and cutaneous mechanical sensitivity, as described previously [8] and separate groups of mice were used to test muscle sensitivity and cutaneous sensitivity. *Muscle mechanical sensitivity* was tested by squeezing the gastrocnemius muscle of the mice with a calibrated pair of tweezers until the mouse withdrew from the stimulus as previously described [10]. The force at which the mouse withdrew was measured in mN. A decrease in threshold was interpreted as muscle hyperalgesia. *Cutaneous mechanical sensitivity* was tested bilaterally by assessing the number of responses to repeated application of a 0.4 mN von Frey filament to the plantar surface of the paw as previously described [23]. The number of withdrawals out of 5 was assessed in 10 trials and an average of all 10 trials was determined for each time period. A significant increase in the number of responses was interpreted as cutaneous hyperalgesia. 

### 2.7. Electron Microscopy

Twenty-four hours after injection of acidic saline, mice were deeply anesthetized sodium pentobarbital (60 mg/kg, i.p.) and transcardially perfused with 2.5% gluteraldehyde in 0.1 M cacodylate buffer. The tibial nerve was dissected bilaterally and placed in the same fixative until processing. Post fixation was carried out for 1 hour at room temperature with a buffered 1% osmium tetroxide solution reduced with 1.5% potassium ferrocyanide. Samples were stained with 2.5% uranyl acetate. Blocks were then rinsed and dehydrated using gradually increasing concentrations of acetone to 100%. Infiltration of Spurr's epoxy resin and acetone were carried out over several days to 100% resin and cured 48 hours in a 60°C oven. Sections of 100 nm thickness were cut using a Leica UC-6 ultramicrotome and collected on 400 mesh copper grids. The grids were then counterstained with 5% uranyl acetate for 2 minutes and Reynold's lead citrate for 2 minutes. Samples were imaged using a JEOL 1230 transmission electron microscope at 120 KV. 

### 2.8. Experimental Design

Experiment 1 tested pH currents in DRG isolated from five groups of mice were used for the present study as follows: a control group that did not receive injections into the gastrocnemius muscle (*n* = 9 mice; 57 cells), a control group injected with pH 7.2 saline once (*n* = 5 mice, 28 cells), an experimental group injected with pH 4.0 saline once (*n* = 4 mice, 22 cells), a control group injected with pH 7.2 saline twice (*n* = 6 mice, n-48 cells), and an experimental group injected with pH 4.0 saline twice (*n* = 6 mice, 41 cells). 

Experiment 2 tested the effects of blockade of ASIC channels, with A-317567, after the development of hyperalgesia. We tested the responses in two separate groups of animals. One group was tested for paw sensitivity (*n* = 6 A-317567, *n* = 6 vehicle) and one for muscle sensitivity (*n* = 6 A-317567, *n* = 6 vehicle). A-317567 was tested against a vehicle control with the tester blinded to drug or vehicle injection. 

Experiment 3 tested if pH 4.0 saline produced nerve damage using electron microscopy in 2 mice per group 24 hours after injections as follows: (1) pH 4.0, single injection, (2) pH 7.2, single injection, (3) pH 4.0 two injections, (4) pH 7.2 two injections. 

### 2.9. Statistical Analysis

Patch clamp data were analyzed using Clampfit (Axon instruments), Microsoft Excel, and Origin 7 software (Northampton MA). A two-way ANOVA was used to study differences between groups and differences between pH using SPSS 17. Post hoc testing between groups was performed with a Tukey's test. *P* < 0.05 was considered significant. Behavioral data were analyzed with a repeated measures ANOVA for differences across time and between groups. Data are represented as mean ± SEM. 

## 3. Results

### 3.1. Injection of pH 4.0 Does Not Alter the Number of DRG Neurons Expressing ASIC-Like Currents

**Figure 1:**
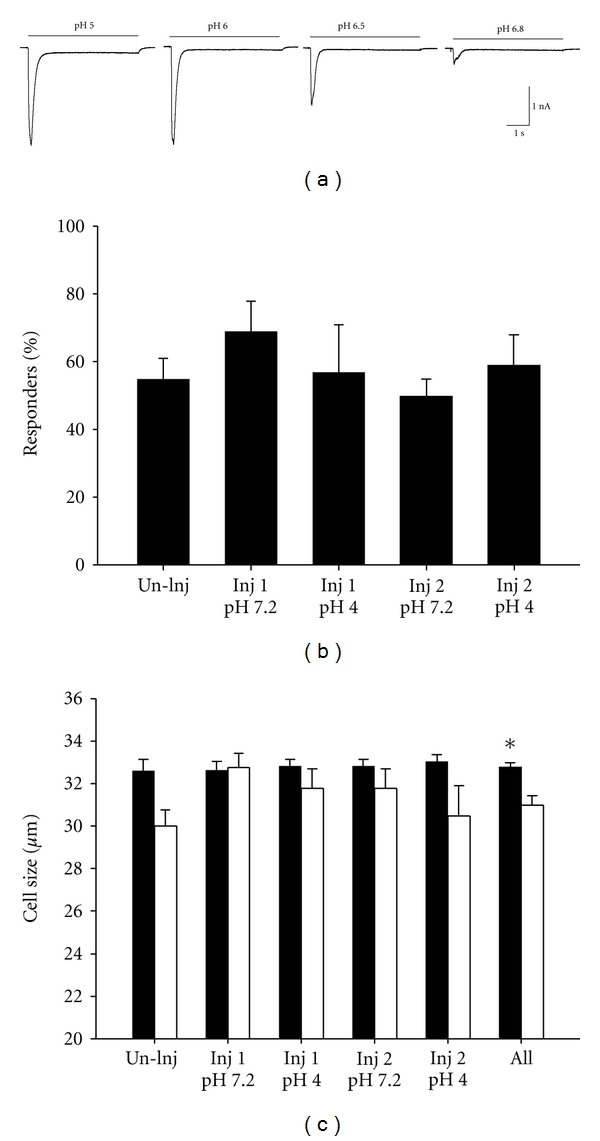
(a) Representative traces of pH currents recorded in DRG neurons innervating muscle. (b) The percentage of labeled muscle DRG neurons that responded to pH 5 application with a transient inward current (ASIC-like current) greater than 100 pA is shown for each experimental group. (c) The mean cell diameters of the muscle DRG neurons that responded to pH 5 application with a transient ASIC-like current (responders; black bars) are compared to those that did not respond (nonresponders; open bars) for each of the experimental groups. The number of responders in each group is as follows: uninjected = 29/57; Injection 1, pH 7.2 = 19/29; Injection 1, pH 4.0 = 22/35; Injection 2, pH 7.2 = 25/48; Injection 2, pH 4.0 = 24/36. *P < 0.05.

To test if ASICs current properties were altered after repeated acid injections, we performed whole-cell patch clamp of retrogradely labeled muscle afferents 24 hours after the second acid injection.  Figure 1(a) shows representative traces of proton-activated currents recorded from a labeled muscle DRG neuron. Acidic pH evoked a rapidly activating transient current that then desensitized in the continued presence of acid. In some neurons pH 5 and pH 6 activated currents had a sustained current along with a transient component. The transient component properties are characteristic of ASICs [6, 22], whereas the sustained component can also represent activation of transient receptor potential subfamily vanilloid 1 (TRPV1) channels in DRG neurons [24]. We previously demonstrated that the transient component of pH-activated currents in muscle DRG neurons was blocked by the ASIC inhibitor, amiloride, and was unaffected by the TRPV1 inhibitor, capsazepine [18]. Therefore, we defined a neuron as expressing an ASIC-like current if pH 5 evoked a transient inward current of greater than 100 pA. We compared the 5 groups of mice (uninjected, one or two injections of pH 7.2 or 4.0) and found no statistical difference in the percentage of labeled muscle afferents that expressed ASIC-like currents (Figure 1(b)). Thus, intramuscular acid injections do not appear to alter the percentage of muscle afferents that express ASICs.

ASICs are expressed in small- and medium-sized neurons that can respond to noxious stimuli and are also expressed in larger neurons that correspond to low threshold mechanoreceptors [25]. Muscle sensory neurons that possessed ASIC-like currents were medium-size-neurons, and neurons that did not express ASIC-like currents were significantly smaller (Figure 1(c)) (*F*_1,195_ = 11.2, *P* = 0.001; sizes ranged in both groups from 20 to 37 *μ*m). Furthermore, there was no difference in cell sizes between treatment groups, suggesting that intramuscular acid injection did not cause a shift in ASIC expression in neurons of a particular size. 

### 3.2. Mean Current Amplitudes of pH-Evoked Currents Are Unchanged after Intramuscular Injection of Acid

**Figure 2:**
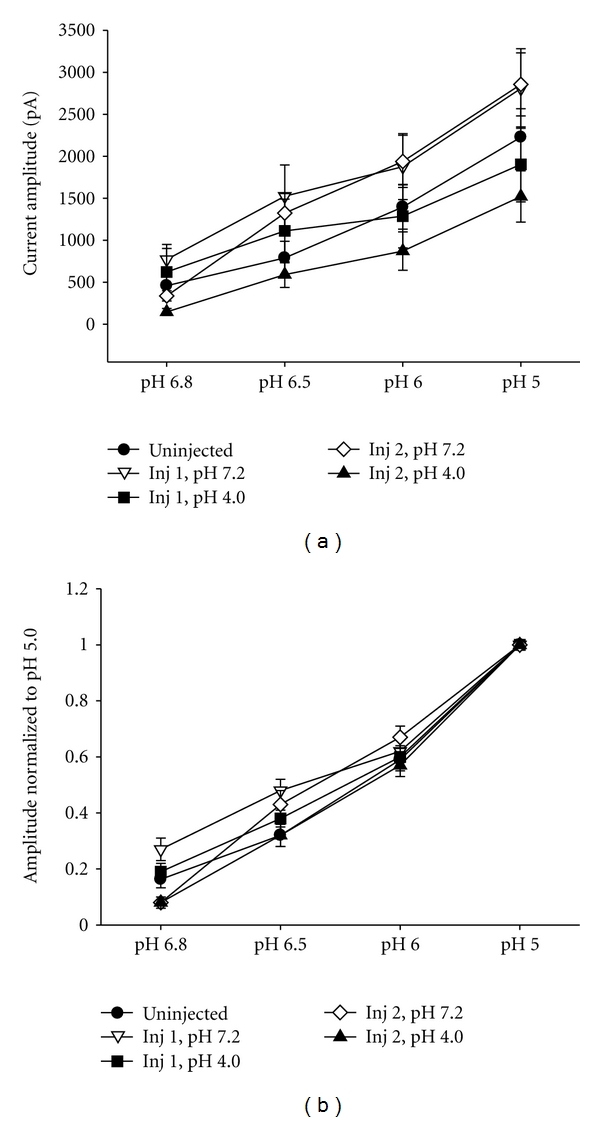
(a) The mean current amplitudes of pH currents at pH 5, 6, 6.5, and 6.8 are not significantly different between groups injected with pH 4.0 (Injection 1, *n* = 22; Injection 2, *n* = 24) and controls injected with pH 7.2 (Injection 1, *n* = 19; Injection 2, *n* = 25), or uninjected controls (*n* = 28). (b) Data from (2A) was normalized to the peak transient currents evoked by pH 5 to analyze pH dose responses. pH dose responses and were not different between groups injection with pH 4.0 (Injection 1, *n* = 21; Injection 2, *n* = 24) and those injected with pH 7.2 (Injection 1, *n* = 19; Injection 2, *n* = 24) or uninjected controls (*n* = 27).

We next examined if there were increases in ASIC expression after inflammation by examining the amplitude of current in response to acidic pH. There was no significant difference between groups for the current amplitude. Twenty-four hours after the first acid injection the mean maximal current amplitude was 1904 ± 446 pA (*n* = 22) (Figure 2(a)) and similar to that after the second acid injection (1521 ± 305, *n* = 24), or pH 7.2 injected controls (Injection 1: 2808 ± 474, *n* = 19; Injection 2: 2856 ± 376, *n* = 25), or uninjected (2228 ± 338, *n* = 28) controls. 

### 3.3. ASIC Channel Properties Were Unaltered by Intramuscular Acid Injections

Sensory neurons have been found to express ASIC1, ASIC2, and ASIC3 isoforms and generally form as heteromers in mouse DRG neurons [6], and each of the different heteromeric combinations of channels displays different biophysical properties [6, 26]. To determine the relative distribution of isoforms we studied different properties of ASIC currents including pH sensitivity of activation, desensitization kinetics, and recovery from desensitization. We hypothesized that hyperalgesia associated with intramuscular acid injections might cause a change in the subunit composition of the ASIC channels, and we could detect this as a change in the biophysical properties.  Figure 2(b) shows that the pH sensitivity of ASIC currents, measured by normalizing the current amplitude recorded at varying pH (6.8–6.0) to the pH 5 current amplitude, did not change significantly after intramuscular acid injections when compared to uninjected and pH 7.2 injected controls. 

**Figure 3:**
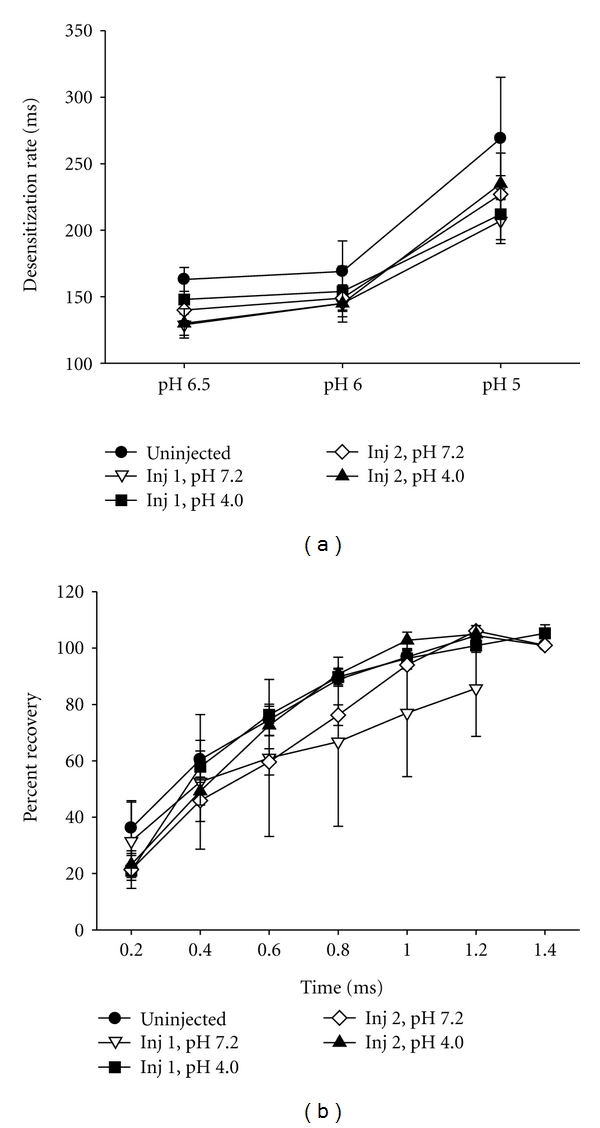
(a) The mean time constants of desensitization of ASIC-like transient currents evoked by the indicated pH solutions were similar between uninjected (*n* = 26), controls injected with pH 7.2 (Injection 1, *n* = 18 Injection 2, *n* = 22) and those injected with pH 4.0 (Injection 1, *n* = 19; Injection 2, *n* = 21). (b) The rate of recovery from desensitization is similar between groups: uninjected (*n* = 12), controls injected with pH 7.2 (Injection 1, *n* = 3; Injection 2, *n* = 15) and those injected with pH 4.0 (Injection 1, *n* = 7; Injection 2, *n* = 10).

ASIC currents desensitize in the continued presence of acidic pH. By fitting the desensitizing phase of the currents to single exponentials, the rates of desensitization (*τ*) were measured.  Figure 3(a) demonstrates that intramuscular acid injections did not change the rate of desensitization of the ASIC-like currents compared to control groups injected with pH 7.2 or uninjected controls. 

After ASIC channels desensitize, they need to be exposed again to a more alkaline pH for some period of time to allow the channels to “recover”, before they can be activated again (see methods for protocol of how recovery was measured).  Figure 3(b) shows that the intramuscular acid injections did not alter the rate of recovery from desensitization. In summary, we found no change in the distribution of muscle DRG neurons that expressed ASIC-like currents, nor were there changes in the current properties at either 24 hours after the first or 24 hours after the second injection of acidic saline. 

### 3.4. Muscle and Cutaneous Hyperalgesia Is Unaffected by Intramuscular Blockade of ASICs with A-317567

**Figure 4:**
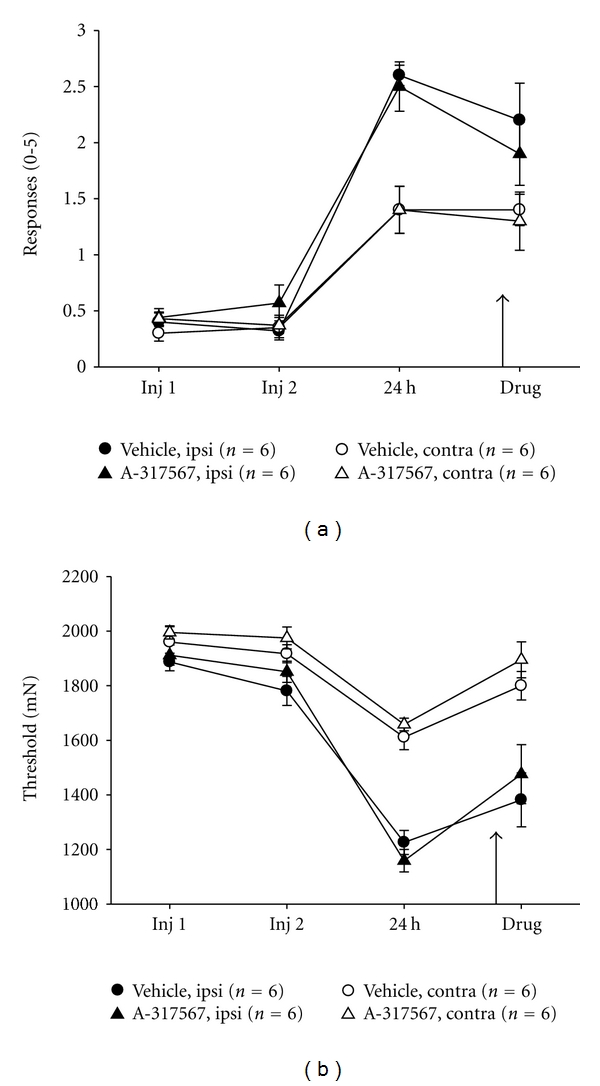
ASIC pharmacological antagonism has no effect upon primary (muscle) or secondary (cutaneous) hyperalgesia induced by repeated intramuscular acid injections. (a) Mean muscle withdrawal thresholds. (b) mean number of responses to repeated von Frey stimulation of the paw measured before first and second acid injections, 24 hours after second acid injection, and after intramuscular injection of A-317567 or control injection. While there was an increase in mechanical sensitivity of the paw and muscle after repeated acid injections, there was no difference in this sensitivity after intramuscular injection of the ASIC antagonist.

Our previous work demonstrated that ASICs are required for the development of secondary hyperalgesia of the paw after repeated intramuscular acid injections [23]. Here we tested if continued activation of ASICs was necessary to maintain the hyperalgesia after it had been developed. As previously shown, repeated intramuscular injection of pH 4.0 saline increases the number of responses of the paw to repeated stimulation (Figure 4(a)) and decreases the force threshold to withdrawal of the muscle (Figure 4(b)), 24 hours after the second injection. Interestingly, intramuscular injection of 0.025 *μ*mol of A-317567, a dose previously shown to reverse muscle cutaneous sensitivity after muscle inflammation [10], had no effect on the enhanced cutaneous and muscle sensitivity induced by repeated acid injections (Figures 4(a) and 4(b)). 

### 3.5. Repeated Acid Injections Do Not Produce Damage to Tibial Nerve

**Figure 5:**
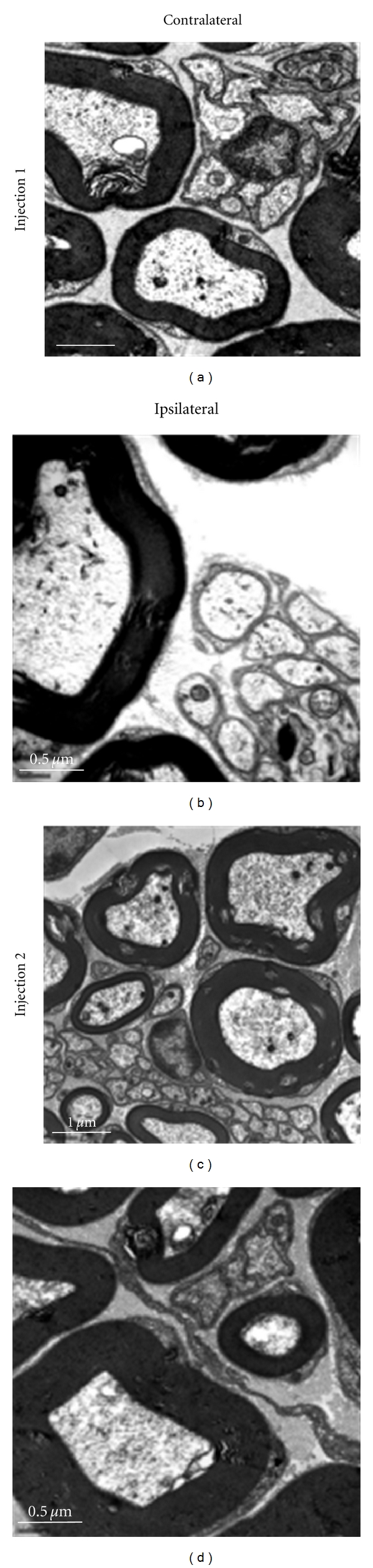
Electron micrograph showing the tibial nerve ipsilateral and contralateral to the site of injection of pH 4.0 saline. The tibial nerve was examined 24 hours after the first injection and 24 hours after the second injection for potential structural changes and inflammatory cell infiltration. There were no observable changes at either time point when compared to the contralateral hindlimb after repeated acid injections.

To be certain that repeated acid injections do not produce damage to the tibial nerve, and hence cause neuropathic pain, we performed electromicrographic analysis of the tibial nerve after injections of pH 4.0 compared to the tibial nerves from the contralateral hindlimb. As shown in Figure 5, there was no difference between the muscles injected with pH 4.0 and those injected with pH 7.2, or from the contralateral hindlimb. This was observed 24 hours after a single injection of pH 4.0 or 24 hours after the second injection of pH 4.0.

## 4. Discussion

We previously found that ASICs are necessary for the development of hyperalgesia after repeated intramuscular acid injection [23]. The current study shows that ASICs are not involved in maintaining the hyperalgesia once it has already developed. We found that pharmacological inhibition of ASICs 24 hours after a second intramuscular acid injection, at a time when hyperalgesia is well established, had no effect upon muscle or cutaneous hyperalgesia. Moreover, we found that repeated acid injections produced no change in the expression of ASIC-like currents or their properties in labeled muscle DRG neurons, suggesting that hyperalgesia in this model is not associated with changes in ASIC expression. 

Prior studies from our laboratory show that ASIC3 is important for the induction of long-lasting mechanical hyperalgesia of the paw after repeated acid injections [23]. Specifically, ASIC3−/− mice do not develop mechanical hyperalgesia of the paw after repeated acid injections when compared to ASIC3+/+ mice [23]. Further, nonselective inhibition of ASICs, or selective blockade of ASIC3, given at the time of the second acid injection prevents the onset of hyperalgesia 24 hours later [23, 27]. This lack of hyperalgesia in ASIC3−/− mice is likely the result of a loss of central sensitization. Recordings from dorsal horn neurons show that ASIC3−/− mice do not show enhanced responsiveness to mechanical stimulation or expansion of receptive fields, measures of central sensitization, when compared to ASIC3+/+ mice [23]. These data, therefore suggest that ASIC3 is important in the induction of long-lasting hyperalgesia and central sensitization. 

This noninflammatory pain model is unique and, once developed, likely depends on central mechanisms. Prior studies from our laboratory and others show that pharmacologically NSAIDs are ineffective while channel blockers and agents with central actions, opioids and pregabalin, reverse the hyperalgesia once developed [28–30]. After the second acid injection, we show that receptive fields of dorsal horn neurons expand to include the contralateral hindlimb, there is increased mechanical sensitivity of the paw bilaterally [23] and Miranda et al., show increased visceral sensitivity [31], all indicative of central changes. Mechanistically, we show an increase in glutamate release both spinally and supaspinally in response to the second, but not the first, intramuscular acid injection [32, 33]. Similarly, we show that local anesthetic blockade at the level of the rostral ventromedial medulla during the second, but not the first, acid injection prevents the development of hyperaglesia 24 hours later [34]. Together, these data suggest that induction of the hyperalgesia initially involves activation of ASICs in muscle afferents, that then send input to the central nervous system to result in enhanced excitability both spinally and supraspinally. 

Once the hyperalgesia develops, however, local anesthetic or neurotrophin-3 delivered directly to the muscle has no effect [22, 35] suggesting that peripheral mechanisms do not maintain the hyperalgesia. Our data are consistent with the conclusion that peripheral mechanisms do not maintain the hyperalgesia since blockade of ASICs did not affect the ongoing hyperalgesia. Similarly, the selective ASIC3 antagonist APETx2 also has no effect on the hyperalgesia in this model once developed [27]. On the other hand, our laboratory showed that local anesthetic blockade supraspinally or blockade of NMDA receptors spinally and supraspinally reverses the hyperalgesia once developed [34, 36, 37]. This activation of NMDA receptors likely turns on second messenger systems to result in long-lasting sensitivity and hyperalgesia. In support we show an increase in phosphorylation of the transcription factor CREB and blockade of the cAMP pathway reverses the hyperalgesia once developed [38]. Thus, once the hyperalgesia develops in the noninflammatory muscle pain model, there are no changes in the number or sensitivity of ASICs and blockade of ASICs has no effect on hyperalgesia.

In contrast inflammatory muscle pain clearly involves activation of ASICs for the maintenance of the hyperalgesia. Specifically, we show that pharmacological blockade of ASICs with the nonselective antagonist A-317567 reverses the hyperalgesia once developed [10]. In both muscle and paw inflammation models, we and others show an increase in the amplitude of pH responsiveness of DRG and an increase in mRNA for ASICs [10, 14]. Our laboratory also showed an increase in the number of joint afferents that express ASIC3 after joint inflammation [8]. This suggests that mechanisms underlying the maintenance of muscle inflammatory pain are uniquely different from those underlying the maintenance of noninflammatory muscle pain. 

The hyperalgesia associated with the muscle is processed differently from that associated with the skin. For ASICs there is a greater expression of ASIC3 in small DRG neurons innervating muscle when compared to DRG neurons innervating skin [39]. In this noninflammatory model, fos expression in response to the muscle stimulation after the development of hyperalgesia increased in the superficial dorsal horn compared to controls and was increased after paw stimulation in acid-injected WT but not acid-injected NT-3 muscle-specific knockouts or NT-3 muscle-injected mice [35, 40]. The lack of injury to the tibial nerve shown in the current study after repeated acid injections is consistent with the fact that there is no peripheral tissue damage or inflammation in response to repeated acid injections [22]. Thus, the muscle hyperalgesia at the site of acid injection and in the contralateral muscle can be considered secondary hyperalgesia. Without peripheral damage to the nerve innervating the paw, we further conclude that the hyperalgesia of the paw is also secondary hyperalgesia.

It is unclear how repeated injections of acidic saline result in widespread hyperalgesia after the second but not the first injection. In addition to muscle and cutaneous hyperalgesia, this model is also associated with enhanced visceral hyperalgesia [31]. The widespread nature of the hyperalgesia suggests involvement in the central nervous system. Peles et al. show that a single injection of pH 4.0 saline enhances spontaneous firing of spinal neurons as well as the response to colorectal distension [41]. It is possible that this sensitization lasts for several days setting up the nervous system to respond in an exaggerated manner to the second acid injection. Indeed both spinally and supraspinally there is enhanced glutamate release during the second acid injection [32, 33] and sensitization of dorsal horn neurons in response to the second acid injection [23]. The increased glutamate at the spinal level is clearly important in the development of the hyperalgesia as spinal blockade of NMDA receptors during the second acid injection delays the onset of hyperalgesia [36]. Thus, the central sensitization induced by peripheral acid injections likely underlies the widespread nature of the hyperalgesia induced by this model. Activation of ASICs, in particular ASIC3, plays a critical role in the development of this central sensitization, which is absent in ASIC3−/− mice [23].

## 5. Conclusion

In summary, the current study shows that once developed the hyperalgesia associated with repeated acid injection does not depend on continued activation of ASICs. There are no changes in expression of ASIC channels in terms of the number or subunit composition and blockade of ASICs after development of hyperalgesia is ineffective. These data further support existing studies showing that the hyperalgesia induced by repeated acid injections is maintained by central mechanisms. 

## References

[1] Waldmann R, Lazdunski M (1998). H^+^-gated cation channels: neuronal acid sensors in the NaC/DEG family of ion channels. *Current Opinion in Neurobiology*.

[2] Reeh PW, Steen KH (1996). Tissue acidosis in nociception and pain. *Progress in Brain Research*.

[3] Frey Law LA, Sluka KA, McMullen T, Lee J, Arendt-Nielsen L, Graven-Nielsen T (2008). Acidic buffer induced muscle pain evokes referred pain and mechanical hyperalgesia in humans. *Pain*.

[4] Sluka KA, Winter OC, Wemmie JA (2009). Acid-sensing ion channels: a new target for pain and CNS diseases. *Current Opinion in Drug Discovery and Development*.

[5] Jasti J, Furukawa H, Gonzales EB, Gouaux E (2007). Structure of acid-sensing ion channel 1 at 1.9 A resolution and low pH. *Nature*.

[6] Benson CJ, Xie J, Wemmie JA (2002). Heteromultimers of DEG/ENaC subunits form H^+^-gated channels in mouse sensory neurons. *Proceedings of the National Academy of Sciences of the United States of America*.

[7] Sluka KA, Radhakrishnan R, Benson CJ (2007). ASIC3 in muscle mediates mechanical, but not heat, hyperalgesia associated with muscle inflammation. *Pain*.

[8] Ikeuchi M, Kolker SJ, Sluka KA (2009). Acid-sensing ion channel 3 expression in mouse knee joint afferents and effects of carrageenan-induced arthritis. *Journal of Pain*.

[9] Ikeuchi M, Kolker SJ, Burnes LA, Walder RY, Sluka KA (2008). Role of ASIC3 in the primary and secondary hyperalgesia produced by joint inflammation in mice. *Pain*.

[10] Walder RY, Rasmussen LA, Rainier JD, Light AR, Wemmie JA, Sluka KA (2010). ASIC1 and ASIC3 play different roles in the development of hyperalgesia after inflammatory muscle injury. *Journal of Pain*.

[11] Price MP, McIlwrath SL, Xie J (2001). The DRASIC cation channel contributes to the detection of cutaneous touch and acid stimuli in mice. *Neuron*.

[12] Chen CC, Zimmer A, Sun WH, Hall J, Brownstein MJ, Zimmer A (2002). A role for ASIC3 in the modulation of high-intensity pain stimuli. *Proceedings of the National Academy of Sciences of the United States of America*.

[13] Staniland AA, McMahon SB (2009). Mice lacking acid-sensing ion channels (ASIC) 1 or 2, but not ASIC3, show increased pain behaviour in the formalin test. *European Journal of Pain*.

[14] Deval E, Noël J, Lay N (2008). ASIC3, a sensor of acidic and primary inflammatory pain. *EMBO Journal*.

[15] Mamet J, Baron A, Lazdunski M, Voilley N (2002). Proinflammatory mediators, stimulators of sensory neuron excitability via the expression of acid-sensing ion channels. *Journal of Neuroscience*.

[16] Ohtori S, Inoue G, Koshi T (2006). Up-regulation of acid-sensing ion channel 3 in dorsal root ganglion neurons following application of nucleus pulposus on nerve root in rats. *Spine*.

[17] Uchiyama Y, Cheng CC, Danielson KG (2007). Expression of Acid-Sensing Ion channel 3 (ASIC3) in nucleus pulposus cells of the intervertebral disc is regulated by p75NTR and ERK signaling. *Journal of Bone and Mineral Research*.

[18] Voilley N, de Weille J, Mamet J, Lazdunski M (2001). Nonsteroid anti-inflammatory drugs inhibit both the activity and the inflammation-induced expression of acid-sensing ion channels in nociceptors. *Journal of Neuroscience*.

[19] Gautam M, Benson CJ, Sluka KA (2010). Increased response of muscle sensory neurons to decreases in pH after muscle inflammation. *Neuroscience*.

[20] Sluka KA, Price MP, Wemmie JA, Welsh MJ, Dostrovsky JO, Carr DB, Koltzenburg M ASIC3, but not ASIC1, channels are involved in the development of chronic muscle pain.

[21] Dubé GR, Lehto SG, Breese NM (2005). Electrophysiological and in vivo characterization of A-317567, a novel blocker of acid sensing ion channels. *Pain*.

[22] Sluka KA, Kalra A, Moore SA (2001). Unilateral intramuscular injections of acidic saline produce a bilateral, long-lasting hyperalgesia. *Muscle & Nerve*.

[23] Sluka KA, Price MP, Breese NM, Stucky CL, Wemmie JA, Welsh MJ (2003). Chronic hyperalgesia induced by repeated acid injections in muscle is abolished by the loss of ASIC3, but not ASIC1. *Pain*.

[24] Caterina MJ, Leffler A, Tominaga M, Rosen TA, Levine JD, Julius D (1997). The capsaicin receptor: a heat-activated ion channel in the pain pathway. *Nature*.

[25] Lingueglia E (2007). Acid-sensing ion channels in sensory perception. *Journal of Biological Chemistry*.

[26] Hesselager M, Timmermann DB, Ahring PK (2004). pH dependency and desensitization kinetics of heterologously expressed combinations of acid-sensing ion channel subunits. *Journal of Biological Chemistry*.

[27] Karczewski J, Spencer RH, Garsky VM (2010). Reversal of acid-induced and inflammatory pain by the selective ASIC3 inhibitor, APETx2. *British Journal of Pharmacology*.

[28] Nielsen AN, Mathiesen C, Blackburn-Munro G (2004). Pharmacological characterisation of acid-induced muscle allodynia in rats. *European Journal of Pharmacology*.

[29] Yokoyama T, Audette KM, Sluka KA (2007). Pregabalin Reduces Muscle and Cutaneous Hyperalgesia in Two Models of Chronic Muscle Pain in Rats. *Journal of Pain*.

[30] Sluka KA, Rohlwing JJ, Bussey RA, Eikenberry SA, Wilken JM (2002). Chronic muscle pain induced by repeated acid injection is reversed by spinally administered *μ*- and *δ*-, but not *κ*-, opioid receptor agonists. *Journal of Pharmacology and Experimental Therapeutics*.

[31] Miranda A, Peles S, Rudolph C, Shaker R, Sengupta JN (2004). Altered visceral sensation in response to somatic pain in the rat. *Gastroenterology*.

[32] Skyba DA, Lisi TL, Sluka KA (2005). Excitatory amino acid concentrations increase in the spinal cord dorsal horn after repeated intramuscular injection of acidic saline. *Pain*.

[33] Radhakrishnan R, Sluka KA (2009). Increased glutamate and decreased glycine release in the rostral ventromedial medulla during induction of a pre-clinical model of chronic widespread muscle pain. *Neuroscience Letters*.

[34] Tillu DV, Gebhart GF, Sluka KA (2008). Descending facilitatory pathways from the RVM initiate and maintain bilateral hyperalgesia after muscle insult. *Pain*.

[35] Gandhi R, Ryals JM, Wright DE (2004). Neurotrophin-3 reverses chronic mechanical hyperalgesia induced by intramuscular acid injection. *Journal of Neuroscience*.

[36] Skyba DA, King EW, Sluka KA (2002). Effects of NMDA and non-NMDA ionotropic glutamate receptor antagonists on the development and maintenance of hyperalgesia induced by repeated intramuscular injection of acidic saline. *Pain*.

[37] da Silva LFS, DeSantana JM, Sluka KA (2010). Activation of NMDA receptors in the brainstem, rostral ventromedial medulla, and nucleus reticularis gigantocellularis mediates mechanical hyperalgesia produced by repeated intramuscular injections of acidic saline in rats. *Journal of Pain*.

[38] Hoeger-Bement MK, Sluka KA (2003). Phosphorylation of CREB and mechanical hyperalgesia is reversed by blockade of the cAMP pathway in a time-dependent manner after repeated intramuscular acid injections. *Journal of Neuroscience*.

[39] Molliver DC, Immke DC, Fierro L, Paré M, Rice FL, McCleskey EC (2005). ASIC3, an acid-sensing ion channel, is expressed in metaboreceptive sensory neurons. *Molecular Pain*.

[40] Sharma NK, Ryals JM, Liu H, Liu W, Wright DE (2009). Acidic saline-induced primary and secondary mechanical hyperalgesia in mice. *Journal of Pain*.

[41] Peles S, Petersen J, Aviv R (2003). Enhancement of antral contractions and vagal afferent signaling with synchronized electrical stimulation. *American Journal of Physiology*.

